# Membranes for Gas Separation

**DOI:** 10.3390/membranes11100755

**Published:** 2021-09-30

**Authors:** Zhien Zhang, Alessio Fuoco, Guangwei He

**Affiliations:** 1 Department of Chemical and Biomedical Engineering, West Virginia University, Morgantown, WV 26506, USA; 2 Institute on Membrane Technology, National Research Council of Italy (CNR-ITM), via P. Bucci 17/C, 87036 Rende, CS, Italy; a.fuoco@itm.cnr.it; 3 School of Chemical Engineering and Technology, Tianjin University, Tianjin 300350, China; guangwei@mit.edu; 4 Department of Chemical Engineering, Massachusetts Institute of Technology, Cambridge, MA 02142, USA

Gas separation is of significant importance for many industrial processes including chemical purification, carbon capture, and fuel production. Generally, gas separation is realized by cryogenic distillation, absorption, adsorption, and membrane processes. Among the technologies for gas removal, separation by the employment of membranes is noteworthy due to the high energy efficiency and productivity of this process, as well as the effective integration with plants [1,2]. However, there are still information gaps in the fields of membrane preparation and characterization, scaling up, separation mechanisms, and process optimization.

In this Special Issue in *Membranes*, 11 review and research papers focused on gas separation using membrane technologies in a wide variety of application fields are included. The Special Issue is available online at https://www.mdpi.com/journal/membranes/special_issues/membr_gas_separation. A concise summary of the presented works in this Special Issue is outlined below.

In the study by Etxeberria-Benavides et al. [3], P84 asymmetric hollow fiber membranes with a very thin (~56 nm) defect-free skin were successfully fabricated by fine-tuning the dope composition and spinning parameters using volatile additive (tetrahydrofuran, THF) as key parameters. An extensive experimental and theoretical study of the influence of volatile THF addition on the solubility parameter of the N-methylpyrrolidone/THF solvent mixture was performed. Although THF itself is not a solvent for P84, in a mixture with a good solvent for the polymer, such as *N*-Methyl-2-pyrrolidone (NMP), it can be dissolved at high THF concentrations (NMP/THF ratio > 0.52). The as-spun fibers had a reproducible ideal CO_2_/N_2_ selectivity of 40 and a CO_2_ permeance of 23 GPU at 35 °C. The fiber production can be scaled up while retaining the selectivity.

In the work by Chouliaras et al. [4], polymeric (ionic liquid) (PIL) copolymers bearing cationic imidazolium pendants and polar acrylic acid groups (P(VBCImY-co-AAx)) were synthesized and blended with film-forming aromatic polyether-based pyridinium PILs (PILPyr) with high glass transition temperature. The copolymers and blends used showed a wide range of glass transition temperatures from 32 to 286 °C, while blends exhibited two-phase morphologies despite the presence of polar groups in the blend components that could participate in specific interactions. Meanwhile, it revealed that blend composition, counter anion type, and acrylic acid molar percentage affected the gas separation properties. In particular, the PILPyr-TFSI/P(VBCImTFSI-co-AA20) blend with 80/20 composition showed CO_2_ permeability of 7 Barrer and a quite high CO_2_/CH_4_ selectivity of 103. Even higher CO_2_/CH_4_ selectivity of 154 was achieved for PILPyr-BF4/P(VBCImBF4-co-AA10) blend with 70/30 composition.

Ghasem [5] develops a two-dimensional mathematical model to predict the chemical absorption of CO_2_ in aqueous methyldiethanolamine (MDEA)-based carbon nanotubes (CNTs) in a hollow fiber membrane (HFM) contactor. The membrane was divided into wet and dry regions, and equations were developed and solved using the finite element method in COSMOL. The results revealed that the existence of solid nanoparticles enhanced the CO_2_ removal rate. The variables with the most significant influence were liquid flow rate and concentration of nanoparticles. Furthermore, there was a good match between experimental and modeling results, with the modeling estimates almost coinciding with experimental data. Solvent enhanced by solid nanoparticles significantly improved the separation performance of the membrane contactor. There was around a 20% increase in CO_2_ removal when 0.5 wt% CNT was added to 5 wt% aqueous MDEA.

Malpass-Evans et al. [6] conducted a systematic study of the differences between four related polymers, highlighting the importance of the role of methyl groups positioned at the bridgehead of ethanoanthracene (EA) and triptycene (Trip) components. The PIMs show BET surface areas between 845 and 1028 m^2^ g^−1^ and complete solubility in chloroform, which allowed for the casting of robust films that provided excellent permselectivities for O_2_/N_2_, CO_2_/N_2_, CO_2_/CH_4_, and H_2_/CH_4_ gas pairs, such that some data surpass the 2008 Robeson upper bounds. Their interesting gas transport properties were mostly ascribed to a combination of high permeability and very strong size-selectivity of the polymers. Time-lag measurements and determination of the gas diffusion coefficient of all polymers revealed that physical aging strongly increased the size-selectivity, making them suitable for the preparation of thin-film composite membranes.

Samarasinghe et al. [7] studied a novel mixed-matrix membrane (MMM) for O_2_/N_2_ separation, which is composed of Matrimid 5218 (Matrimid) as the matrix, cobalt(II) phthalocyanine microparticles (CoPCMPs) as the filler, and Pluronic F-127 (Pluronic) as the compatibilizer. By the incorporation of CoPCMPs with Matrimid but without Pluronic, interfacial defects were formed. Pluronic-treated CoPCMPs, on the other hand, enhanced O_2_ permeability and O_2_/N_2_ selectivity by 64% and 34%, respectively. The enhancement achieved an increase in both O_2_ diffusivity and O_2_/N_2_ solubility selectivity.

Chuah et al. [8] used HKUST-1 in mixed-matrix membrane fabrication to investigate its potential for improving C_2_H_4_/C_2_H_6_ separation performance. Prior to membrane fabrication and gas permeation analysis, nanocrystal HKUST-1 was first synthesized. This step is critical in order to ensure that defect-free mixed-matrix membranes can be formed. Polyimide-based polymers, ODPA-TMPDA and 6FDA-TMPDA, were then chosen as the matrices. It was revealed that 20 wt% loading of HKUST-1 was capable of improving C_2_H_4_ permeability (155% for ODPA-TMPDA and 69% for 6FDA-TMPDA) without excessively sacrificing the C_2_H_4_/C_2_H_6_ selectivity. The C_2_H_4_ and C_2_H_6_ diffusivity and solubility were also improved substantially compared to the pure polymeric membranes. Overall, our results are near the upper bound, confirming the effectiveness of leveraging nanocrystal HKUST-1 filler for performance enhancements in mixed-matrix membranes for C_2_H_4_/C_2_H_6_ separation.

Tysa et al. [9] presented a simple flow-based system for the determination of the preservative agent sulfite in food and beverages. The standard method of conversion of sulfite ions into SO_2_ gas by acidification is employed to separate the sulfite from sample matrices. The sample is aspirated into a donor stream of sulfuric acid. A membrane gas–liquid separation unit, also called a “gas-diffusion (GD)” unit, incorporating a polytetrafluoroethylene (PTFE) hydrophobic membrane, allows the generated gas to diffuse into a stream of deionized water in the acceptor line. The dissolution of the SO_2_ gas leads to a change in the conductivity of water, which is monitored by an in-line capacitively coupled contactless conductivity detector (C4D). The conductivity change is proportional to the concentration of sulfite in the sample. In this work, both clear (wine) and turbid (fruit juice and extracts of dried fruit) were selected to demonstrate the versatility of the developed method. The method can tolerate turbidity up to 60 nephelometric turbidity units (NTUs). The linear range is 5–25 mg/L ${\mathrm{SO}}_{3}^{2-}$ with precision <2% RSD. The flow system employs a peristaltic pump for propelling all liquid lines. Quantitative results of sulfites were statistically comparable to those obtained from iodimetric titration for the wine samples.

Muzzi et al. [10] studied the correlation of the structural features of different membrane materials, analyzed by means of molecular dynamics simulation and their gas diffusivity/selectivity. They proposed a simplified method to determine the void size distribution via an automatic image recognition tool, along with a consolidated Connolly probe to sense space, without the need for demanding computational procedures. Based on a picture of the void shape and width, automatic image recognition tests the dimensions of the void elements, reducing them to ellipses. Comparison of the minor axis of the obtained ellipses with the diameters of the gases yields a qualitative estimation of non-accessible paths in the geometrical arrangement of polymeric chains. A second tool, the Connolly probe to sense space, provides more details on the complexity of voids. The combination of the two proposed tools can be used for qualitative and rapid screening of material models and to estimate the trend in their diffusivity selectivity. The main differences in the structural features of three different classes of polymers are investigated in this work (glassy polymers, superglassy perfluoropolymers and high free-volume polymers of intrinsic microporosity), and the results show how the proposed computationally less demanding analysis can be linked with their selectivities.

Scaling up this technology from laboratory to industry is an unsolved challenge because it requires the improvement of the experimental methodologies that replicate lab-scale results at a larger scale. Quilaqueo et al. [11] compared the performance of three different hollow fiber membrane contactor modules (1.7 × 5.5 mini module, 1.7 × 10 mini module, and 2.5 × 8 extra flow). These are used for recovering cyanide from aqueous solutions at a laboratory scale using identical operational conditions. For each experimental set-up, mass-transfer correlations in the ranges of feed flows assayed were determined. The modules with the smallest and largest area of mass transfer reached similar cyanide recovery rates (>95% at 60 min), which demonstrates the impact of module configuration on their operating performance. The results obtained here are limited to scaling up the membrane module performance only because operating modules with the largest area result in low *Re* numbers. This fact limits the extrapolation of results from the mass-transfer correlation.

Alqaheem and Alomair [12] show the characterization techniques for studying the physical structure such as scanning electron microscopy (SEM), transmission electron microscopy (TEM), and atomic force microscopy (AFM). Techniques for investigating the crystal structure such as X-ray diffraction (XRD), small-angle X-ray scattering (SAXS), and wide-angle X-ray scattering (WAXS) are also considered. Other tools for determining the functional groups, such as Fourier transform infrared spectroscopy (FTIR), Raman spectroscopy and nuclear magnetic resonance (NMR), are reviewed. Methods for determining the elemental composition, such as energy-dispersion X-ray spectroscopy (EDS), X-ray fluorescent (XRF), and X-ray photoelectron spectroscopy (XPS), are explored. The paper also gives general guidelines for sample preparation and data interpretation for each characterization technique.

Li and coworkers [13] reviewed the operating principle and wetting mechanism of hollow membrane contactors, showing the latest research progress of membrane contactors in gas separation, especially for the removal of carbon dioxide from gas mixtures by membrane contactors, and summarized the main aspects of membrane materials, absorbents, and membrane contactor structures. Furthermore, recommendations are provided for the existing deficiencies or unsolved problems (such as membrane wetting), and the status and progress of membrane contactors are discussed.

In conclusion, the editors would like to thank the authors and reviewers for their valuable contributions to this Special Issue and the editorial staff of *Membranes* for their help and support during the review process.

## Data Availability

Not applicable.
